# A Little Dab Will Do: A Case of Cannabis-Induced Psychosis

**DOI:** 10.7759/cureus.10311

**Published:** 2020-09-08

**Authors:** Garrett Rossi, Melanie Beck

**Affiliations:** 1 Psychiatry, Cooper University Hospital, Camden, USA; 2 Psychiatry, Cooper Medical School of Rowan University, Camden, USA

**Keywords:** cannabis use, addiction psychiatry, cl psychiatry, cannabis, marijuana use, psychosis, cannabis-induced psychosis

## Abstract

A patient presented to the emergency department with his brother due to recent onset of paranoid behavior that escalated over the past month. The patient endorsed paranoid delusions of people watching him and following him in a black truck. The patient admitted to being unable to sleep for the past two weeks and to having hypervigilant behavior whenever he leaves the house. Due to the patient’s presentation, the differential diagnosis included schizophrenia, substance-induced psychotic disorder, psychotic disorder due to another medical condition, bipolar disorder, and major depressive disorder with mood incongruent psychotic features. Upon interview, the patient stated he was using marijuana to decrease his self-reported anxiety and other social stressors since the age of 13 years. Over the past month, the patient says he has been “dabbing,” which is a highly concentrated form of cannabis mainly consumed by experienced users. These “dabs” have an extremely high tetrahydrocannabinol (THC) content (up to 80%), which is the main psychoactive component in cannabis products. Since the patient began using this potent form of cannabis, he has had increasing difficulty functioning at work and worsening symptoms of psychosis. After eliciting this information and noting the patient had a positive urine drug screen for cannabis, a diagnosis of cannabis-induced psychosis along with severe cannabis use disorder was made. There is a trend towards increasing THC concentrations in cannabis products. This case highlights the importance of being aware of these highly potent cannabis products and their potential harms. Patients should approach these products with caution, as they are not only more dangerous to manufacture but also have the potential to induce psychosis in susceptible populations.

## Introduction

Marijuana products have greater potency and higher levels of tetrahydrocannabinol (THC) than ever before. THC levels in marijuana products are regularly exceeding 20%, but this is mild in comparison to various types of cannabis concentrates collectively known as “dabs" [1]. These “dabs” have many names, such as honey, hash oil, wax, shatter, and extract. These highly potent marijuana products are created by using an extraction process to isolate the active ingredient THC in the marijuana plant. There are a number of different methods to separate the cannabinoids from the plant, using techniques involving ethanol and carbon dioxide, but one of the increasingly popular processes involves using butane. The cannabis and liquid butane are pressurized and heated into a mixture [2]. Due to the volatility of butane, as the mixture evaporates under a vacuum, the butane turns from liquid to vapor making it easier to remove. This leaves behind crystal resins known as “shatter.” There are few cannabis-based concentrates that are stronger than shatter. This drug is normally reserved for the experienced cannabis user due to the ultra-high THC concentrations, reaching upwards to 80% in some cases [3]. There is very little research regarding the psychiatric consequences of using cannabis products with ultra-high concentrations of THC. In the hands of inexperienced users, there can be serious consequences including increased rates of anxiety, paranoia, addiction, and psychosis. This case report details the course of a patient who experienced cannabis-induced psychosis from the use of “dab.”

## Case presentation

The patient was brought to the emergency department by his older brother for paranoid behavior escalating over the last one month. His brother informed the staff that he had increasing concerns about the patient’s behavior. The patient had been isolated for the past week and was making comments about people watching his house and following him in a black pickup truck. He reported being unable to sleep for the past two weeks and remaining hypervigilant whenever he left the house. His work as a general contractor was suffering as a result of the paranoia, and he felt like he was losing his grip on reality.

His social history was significant for multiple adverse childhood events, including separation of his parents at the age of two years, growing up with a mother who carried a diagnosis of bipolar disorder, and physical abuse by his stepfather. He reported being connected with a therapist as a child, from the ages of 8-14 years, but was never evaluated by a psychiatrist. He was never diagnosed with a psychiatric disorder and had no prior psychiatric hospitalizations. In order to cope with his difficult home life and self-reported chronic anxiety, the patient began using marijuana at the age of 13 years. He has used marijuana on a daily basis (two to three blunts per day) since that time, and has gradually progressed to more potent forms of the drug. The patient stated, “I’ve been dabbing for the past month and he believed it may be contributing to my symptoms.” The patient’s brother went on to explain that “dab” is a highly concentrated form of THC more potent than most recreationally available products. His laboratory studies were unremarkable with the exception of a urine drug screen positive for cannabis. 

His mental status revealed a well-developed, well-nourished Caucasian male who appeared his stated age. He appeared suspicious but was willing to engage in conversation. His mood was “anxious” and his affect was paranoid. His thought process was linear, but his thought content was notable for paranoid delusions about people watching and following him. He denied suicidal or homicidal ideation with intent or plan. He denied auditory or visual hallucinations. His judgment was poor, and his insight was considered to be limited. 

Prior to his presentation, the patient had attended work on a daily basis, and enjoyed spending time with family and friends. The patient had a girlfriend who he recently broke up with due to his continued cannabis use, and recent paranoid behavior. He admitted to accusing her on multiple occasions of talking to other men and having sexual relationships with them. He had no proof to substantiate these accusations, and the timeline was consistent with his increased use of dab. 

He was concerned about his paranoid thoughts, and loss of function at work. He requested voluntary admission to the inpatient psychiatric facility for further monitoring and evaluation. The referral was made, and a bed was available for transfer. He spent five days on the inpatient unit and was treated with 1 mg twice daily of risperidone, which resulted in gradual normalization of his paranoid thoughts and return to baseline functioning. He was followed up in the outpatient clinic and gradually tapered off of risperidone. He was also connected with a substance use counselor, who helped the patient maintain his sobriety. After seven months, the patient was functioning well at work and going back to college. He did not have a relapse of psychotic symptoms, and after one year discontinued psychiatric treatment. 

## Discussion

The extraction process associated with butane hash oil (BHO) has allowed the content of marijuana-based products to drastically change. More potent products containing higher levels of THC have recently become more popular, and new cases are presenting to emergency room facilities daily. As healthcare providers it is important to not only be able to educate our patients about the potential hazards that result from using these products but also make them aware of the dangers associated with manufacturing them.

One of the largest dangers associated with preparing “dab” comes from the use of the extraction device used to make these products. There have been multiple cases of burns reported while attempting to manufacture this product as butane is very flammable and it has to be heated to very high temperature for extraction [4]. Once the product is manufactured, there are still risks associated with the apparatus known as “the oil rig” used to smoke these substances. This apparatus contains a nail that has to be heated to 400 degrees using a blowtorch in order to vaporize the substances for inhalation [2]. The high temperature not only serves as a factor contributing to the increased risk of burns but also allows by-products such as rust and benzene to be produced and inhaled. The most common group of people who use BHO purified products are people already using some form on marijuana [5]. It is important to identify these patients and educate them about the risks of BHO products. It was shown that BHO users in particular were more likely to be of lower education and that patients with bipolar disorder were more likely to use BHO than high-potency herbal cannabis [3]. 

Despite the risks of manufacturing this product, there are also increased risks associated with ingesting this product. Marijuana contains both cannabidiol (CBD) and THC, and it has been shown that the presence of CBD can be protective against psychosis [4]. These BHO products have increased THC concentrations and tend to have lower CBD concentrations; therefore, they put people at a higher risk of developing psychosis. There is also an increased risk of addiction with BHO products resulting in increased tolerance and withdrawal compared to the traditional cannabis plant. A higher level of physiological dependence in BHO users has also been shown, but the mechanism for this remains unclear [5]. Possible mechanisms include THC downregulating the CB1 receptor in the brain, which is responsible for tolerance [6]. Other possibilities include BHO having a shorter half-life leading to severe withdrawal symptoms, or BHO resulting in more positive reinforcement as it takes a smaller amount of this product to achieve the same effects of more traditional marijuana products. Despite the varying theories, the clinical evidence remains clear that there is a higher level of tolerance and withdrawal associated with BHO products.

## Conclusions

It appears that the case reports published on this topic reveal two scenarios. The resolution of psychosis with the use of low-dose atypical antipsychotics or what appears to be a prolonged substance-induced psychosis. This case demonstrated the success of treatment with 1 mg risperidone twice daily. However, there have not been any trials conducted relating to using different antipsychotic medications with psychosis induced from “dab”. Further research is necessary to determine the potential long-term consequences of using high potency THC products purified with BHO as well as to examine why some of the presentations resolve successfully while others continue to experience symptoms. As the use of these types of products continues to increase, it is imperative to not only understand the risks associated with manufacturing and inhaling these products but also to understand the commonalities among cases.
